# Progress of Iron-Based Nanozymes for Antitumor Therapy

**DOI:** 10.3389/fchem.2020.00680

**Published:** 2020-09-10

**Authors:** Linawati Sutrisno, Yan Hu, Yanhua Hou, Kaiyong Cai, Menghuan Li, Zhong Luo

**Affiliations:** ^1^School of Life Science, Chongqing University, Chongqing, China; ^2^Key Laboratory of Biorheological Science and Technology, Ministry of Education, College of Bioengineering, Chongqing University, Chongqing, China; ^3^Chongqing Engineering Research Centre of Pharmaceutical Sciences, Chongqing Medical and Pharmaceutical College, Chongqing, China

**Keywords:** iron-based nanozymes, biocatalysis, nanomedicine, tumor therapy, enzyme mimics

## Abstract

Artificial nanoscale enzyme-mimics (nanozymes) are promising functional alternatives to natural enzymes and have aroused great interest due to their inherent *in vivo* stability, affordability, and high catalytic ability. Iron-based nanozymes are one of the most investigated synthetic nanomaterials with versatile enzyme-like catalytic properties and have demonstrated remarkable relevance to a variety of biomedical applications, especially biocatalytic therapy against tumor indications. Nevertheless, despite the recent advances in biology and nanotechnology, the therapeutic performance of iron-based nanozymes *in vivo* is still limited by technical issues such as low catalytic efficiency and lack of tumor specificity. In this mini review, we briefly summarized the representative studies of iron-based nanozymes, while special emphasis was placed on the current challenges and future direction regarding the therapeutic implementation of iron-based nanozymes for the development of advanced tumor therapies with improved availability and biosafety.

## Introduction

Enzymes are a class of powerful catalysts that are responsible for accelerating various chemical reactions in the human body and are required to promote numerous biological processes, such as metabolism, detoxification, and biosynthesis (DeBerardinis and Chandel, 2016; Huang et al., 2019; Leveson-Gower et al., 2019; Wu et al., 2019). Owing to their potential effect on catalyzing the chemical reaction, enzymes have been exploited to inhibit tumor proliferation. However, the clinical translation of the enzyme is limited by its susceptibility to environmental stress as well as manufacturing problems including difficult synthesis and high production costs. Thanks to the recent advances in nanotechnology and enzymology, artificial enzymes (nanozymes) have been developed for various biomedical applications due to their tunable catalytic activities, affordable cost, facile synthesis, and high structural stability (Gao and Yan, 2016). Iron-based nanozymes (INs) are one of the earliest inorganic nanomaterials with exploitable catalytic behaviors (Cramer and Kampe, 1965). Some representative examples are ferromagnetic nanoparticles and Prussian blue (PB) nanoparticles, which may provide critical benefits for tumor treatment including Fenton-augmented ROS stress and hypoxia amelioration. These promising features of iron-based nanozymes have thus inspired great scientific interest for therapeutic intervention against a variety of tumor indications.

Nevertheless, the clinical implementation of iron-based nanozymes has met with several challenges. Typically, these nanozymes are prone to aggregation and biofouling in the biological microenvironment, both of which could lead to reduced catalytic efficiency. Moreover, the mechanisms underlying the catalytic activity of INs are still elusive and their recycling and regeneration *in vivo* remain a major challenge. In this review, we will first provide a brief summary of the previous clinical exploitations of INs for cancer therapy and then special emphasis will be placed on those major impacting factors affecting their catalytic efficiency and therapeutic performance, as well as representative studies to overcome these limitations. A perspective on the possible breakthroughs in future nanozyme-mediated tumor therapies is also provided, which may offer indicative insight for research in related areas.

## Reaction Routes Underlying the Catalytic Activity of Representative INs

As described above, iron-based nanomaterials are some of the most promising nanodrugs. They have been applied first to translational studies and several iron-containing nanoformulations have been approved by the FDA for clinical usage. Typically, INs have demonstrated many clinically favorable properties that are particularly relevant for tumor therapy. From a physiochemical perspective, previous study indicates that iron-based nanoparticles could remain stable even after being stored for 40 days at ambient temperature, and their catalytic efficiency is still maintained after multiple treatment sessions (Zhang et al., 2009; Woo et al., 2013). INs also have excellent biocompatibility, which contributes to both the efficacy and safety of the IN-mediated treatment. In addition, INs could efficiently be deposited into tumor tissues based on their controllable magnetic properties to increase the therapeutic index. Moreover, INs are more resistant to environmental stress such as basic/acidic environments and extreme temperatures compared to natural enzymes. Furthermore, INs have excellent morphological homogeneity and are easy to produce at an affordable price. Based on the advantages described above, INs contributed significantly to the advances of nanocatalytic cancer therapy.

Recent insights collectively demonstrated that the biocatalytic activity of iron-based nanozymes is strongly affected by the pH of the ambient environment. Specifically, under acidic pH some INs possess peroxidase-mimicking abilities where they could react with the excessive hydrogen peroxide (H_2_O_2_) in cancer cells to produce hydroxyl radicals via a Fenton reaction, which have higher reactivity than normal ROS and can amplify the cytotoxic damage to the tumor cells (Bokare and Choi, 2014; Ranji-Burachaloo et al., 2018). Alternatively, under the neutral pH in tumor cytosol, some INs could demonstrate catalase-like activity and decompose H_2_O_2_ into oxygen and water, which has particular relevance for the treatment of hypoxic tumors (Lee et al., 2011; Abdollah et al., 2018). The chemical reactions underlying these two enzyme-mimicking abilities are described below.

Peroxidase-mimicking abilities of INs under acidic condition (Li et al., 2020):

(1) $$\begin{array}{l} {\text{Fe}}^{2+}+{\text{H}}_{2}{\text{O}}_{2}\to {\text{Fe}}^{3+}+{\text{HO}}^{•}+{\text{OH}}^{-} \end{array}$$

(2) $$\begin{array}{l} \text{RH}+{\text{HO}}^{•}\to {\text{R}}^{•}+{\text{H}}_{2}\text{O} \end{array}$$

(3) $$\begin{array}{l} {\text{R}}^{•}+{\text{Fe}}^{3+}\to {\text{Fe}}^{2+}+\text{non}-\text{radical product }\left(\text{termination}\right) \end{array}$$

(4) $$\begin{array}{l} {\text{H}}_{2}{\text{O}}_{2}+{\text{HO}}^{-}\to -{\text{HO}}_{2}+{\text{H}}_{2}\text{O} \end{array}$$

(5) $$\begin{array}{l} {\text{Fe}}^{2+}+{\text{HO}}^{•}\to {\text{Fe}}^{3+}+{\text{HO}}^{-} \end{array}$$

The peroxidase-like catalytic property of INs usually originates from the heme-like structures therein, although the detailed mechanism is still not clear. Heme is a prosthetic group that consists of an iron atom in the center of a large porphyrin ring, which is found in both peroxidase and catalase. In this reaction process, H_2_O_2_ is converted to hydroxyl radicals as the intermediate products, and then the hydroxyl radicals capture protons from the hydrogen donor, forming water and oxidized donor. To achieve the optimal peroxidase-mimicking activity of the INs, the temperature must be in the range of 37–40°C and the pH should be in the range of 3–6.5. Previous study also reported that both Fe_2_O_3_ and Fe_3_O_4_ could demonstrate peroxidase-mimetic activity, wherein the activity of the former is better (Chen et al., 2012). INs can also be stimulated by some reported activators, including adenosine triphosphate (ATP), adenosine diphosphate (ADP), and adenosine monophosphate (AMP). Notably, ATP can improve the peroxidase-mimicking activity under neutral pH by interfering with single electron transfer reactions (Gao et al., 2017).

Catalase-mimicking activities of INs under neutral or basic pH:

(6) $$\begin{array}{l} {\text{Fe}}^{3+}+{\text{H}}_{2}{\text{O}}_{2}\to {\text{FeOOH}}^{2+}+{\text{H}}_{2}\text{O} \end{array}$$

(7) $$\begin{array}{l} \text{FeOO}{\text{H}}^{2+}\to {\text{Fe}}^{2+}+{\text{HO}}_{2}^{•} \end{array}$$

(8) $$\begin{array}{l} {\text{Fe}}^{3+}+{\text{H}}_{2}{\text{O}}_{2}\to {\text{Fe}}^{3+}+{\text{HO}}^{-}+{\text{OH}}^{•} \end{array}$$

(9) $$\begin{array}{l} {\text{HO}}_{2}^{•}\to {\text{H}}^{+}+{\text{O}}_{2}^{-} \end{array}$$

(10) $$\begin{array}{l} {\text{OH}}^{•}+{\text{HO}}_{2}^{•}/{\text{O}}_{2}^{-}\to {\text{H}}_{2}\text{O}+{\text{O}}_{2} \end{array}$$

As for catalase-mimicking properties, it is well-established that INs effectively catalyze the degradation of H_2_O_2_ into water and oxygen, which could be used to relieve the hypoxia condition in the tumor microenvironment (Li et al., 2020). Gu et al. first compared the catalase-mimicking biocatalytic properties between Fe_3_O_4_ and Fe_2_O_3_ and reported that Fe_3_O_4_ demonstrated a higher catalytic ability than Fe_2_O_3_ (Wei and Wang, 2008). Based on these underlying mechanisms, scientists have developed a plethora of iron-containing nanoformulations for tumor therapy, which will be briefly discussed below individually.

## INs for Nanocatalytic Cancer Therapy

As demonstrated by the mechanistic discussions above, INs could demonstrate multifaceted catalytic activities resembling various enzymes *in vivo*. This, in combination with the magnetic responsiveness, versatile surface chemistry, and excellent biocompatibility of iron-based nanomaterials, INs have been increasingly applied for the diagnosis and treatment of many tumor indications. For instance, Cai et al. demonstrated that the iron core in the ferrimagnetic H-ferritin nanoparticles had peroxidase-like catalytic activities and could be used for the immunohistochemical-like staining of the tumor tissues. The authors observed that xenografted tumor tissues incubated with the ferrimagnetic H-ferritin nanoparticles showed a brownish color due to the nanozyme-catalyzed oxidation of 3,3'-diaminobenzidine tetrahydrochloride substrates under the presence of excessive H_2_O_2_, while the normal tissues were stained purple by hematoxylin (Cai et al., 2015). Meanwhile, it has also been reported that catalase-like INs could decompose the tumor H_2_O_2_ to generate additional oxygen, which could be exploited to facilitate the ultrasound imaging of the tumor area (Wang et al., 2020). As for the therapeutic exploitation of the INs, current research mostly focuses on the application as Fenton nanocatalysts or oxygen generators, which will be discussed in the section below.

### Nanocatalysts Based on Iron Oxide Nanostructures

Superparamagnetic iron oxide nanoparticles (SPIONs) are a class of biocompatible and degradable inorganic nanomaterials that have been widely explored for tumor diagnosis and therapy, which refers to small nanocrystals composed of iron oxide (usually in the form of magnetite Fe_3_O_4_ or maghemite γ-Fe_2_O_3_). After being internalized by cancer cells, SPIONs could demonstrate multifaceted catalytic functions for a variety of applications. For instance, SPIONs display peroxidase-mimetic properties via a Fenton reaction under an acidic microenvironment, while they are also capable of decomposing H_2_O_2_ under the neutral and basic pH conditions showing catalase-mimetic activity. Hence, the nanocatalytic effect of the SPIONs is dependent on their local pH, which could be exploited to modulate the therapeutic activities in biological environments (Chen et al., 2012).

Typically, it has been reported that ferumoxytol, an FDA-approved intravenous iron preparation based on SPIONs coated with lower molecular weight semi-synthetic carbohydrates, exhibits nanocatalytic therapeutic effects on leukemia cells with low ferroportin levels (Zanganeh et al., 2016). It was further revealed that ferumoxytol generates ROS at higher rates than free iron nanoparticles and the ROS production rate could remain at a steady level for a long time. Since the leukemic cells with low ferroportin expression levels are unable to efficiently export the ferumoxytol nanoformulations, the ROS generation cannot be stopped via normal antioxidation mechanisms, eventually leading to cell death. On the contrary, normal cells have high ferroportin expression, allowing the cells to export the exogenous iron species after ferumoxytol uptake and ameliorate the ferumoxytol-mediated Fenton-based cytotoxic damage (Trujillo-Alonso et al., 2019).

**Figure 1:**
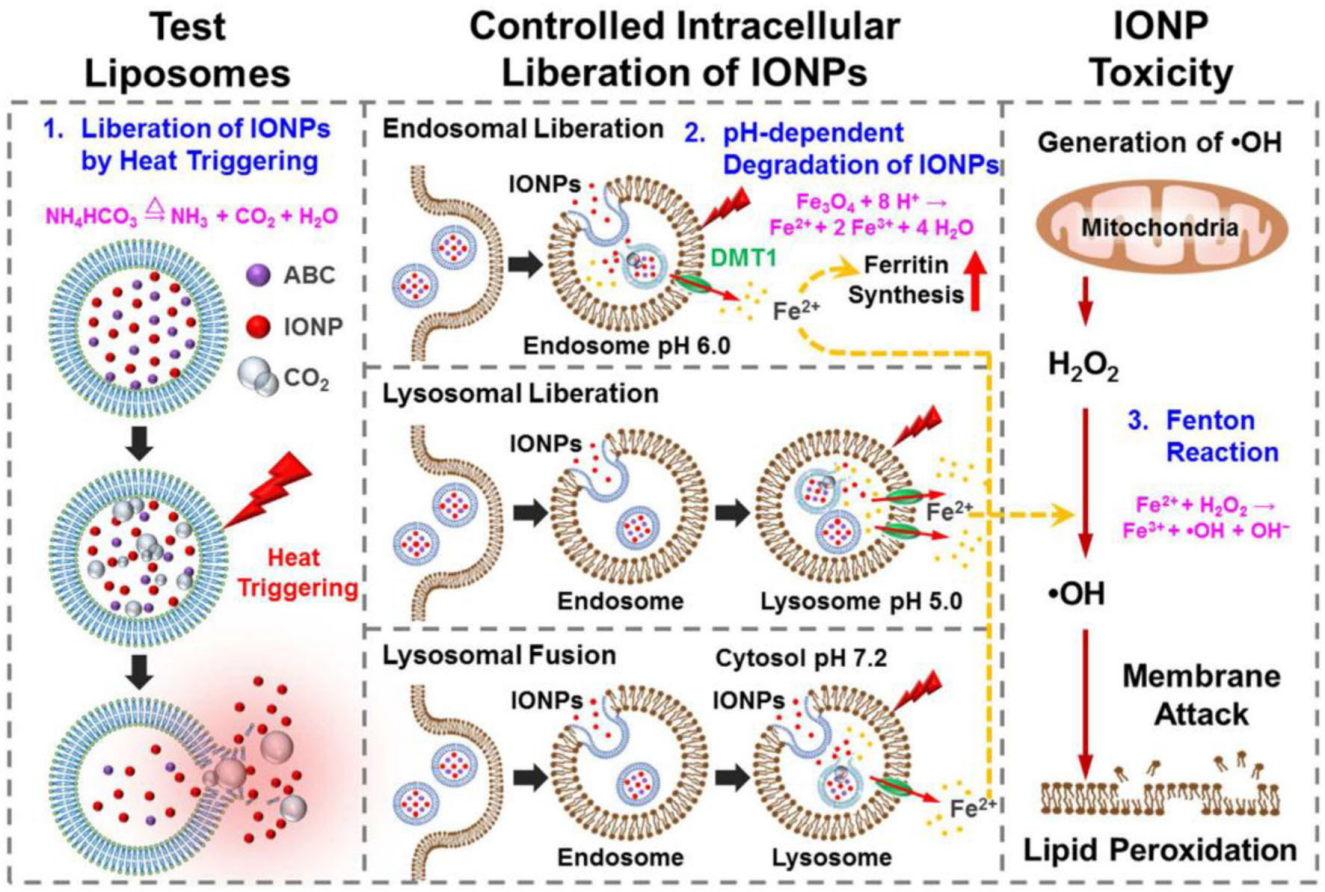
Schematic illustrations of the structure of thermoresponsive bubble-generating liposomal system and its process of spatially precise versatile nanosystem-controlled intracellular liberation of IONPs in specific cellular organelles in various endocytic stages. The degradation of IONPs, release of iron ions, and subsequent reactive oxygen species (ROS) generation within cells are indicated. IONPs, iron oxide nanoparticles; ABC, ammonium bicarbonate; DMT1, divalent metal transporter-1. Reproduced with permission from Huang et al. (2016). Copyright © 2016, American Chemical Society.

It's also possible to regulate the catalysis-based cancer therapy of SPION-based INs by combining them with other nanobiomaterials. For instance, Huang and coworkers fabricated a bubble-generating liposomal system for the delivery of iron oxide-based nanozymes, which was also loaded with ammonium bicarbonate to trigger the release of the INs in a responsive manner (Huang et al., 2016; Figure 1). After being internalized by cancer cells, ammonium bicarbonate would be hydrolyzed into CO_2_ and NH_3_ in the acidic environment of the endo/lysosomes, thus disrupting the liposomal membrane and releasing the INs, which would then react with the excessive H_2_O_2_ in the tumor cytosol and produce large amounts of cytotoxic hydroxyl radicals. The proposed controlling mechanism was also supported by the minimal toxicity of the nanosystem in early endosomes and the pronounced cytotoxic damage after reaching the cytoplasm. This study further confirmed the possibility of the acidity for controlling the Fenton reaction in the intracellular environment for amplifying the therapeutic efficacy.

### Other Types of Iron-Containing Nanostructures With Catalytic Activity

Aside from the iron oxide nanostructure, some other iron-containing nanostructures have also been widely explored like a nanocatalytic therapeutic nanostructure used for cancer treatment. Prussian Blue (Fe_4_[Fe(CN)_6_]_3_, PB) has been used in various biomedicine fields due to its multi-enzyme-like capabilities, including peroxidase, catalase, and superoxide dismutase. This phenomenon could be explained by the abundant redox potential of the various constituents of PB, which makes it an efficient electron transporter (Zhang et al., 2016). The enzyme-like catalytic mechanism of PB is distinctively different from conventional Fenton reactions, such as the capability of scavenging hydroxyl radicals due to their high affinity. The peroxidase-mimetic reaction routes of PB is demonstrated here below:

$$\begin{array}{l} \left[\text{Fe}\left(\text{III}\right)\text{Fe}\left(\text{II}\right){\left(\text{CN}\right)}_{6}\right]-\left(\text{PB}\right)\\ {\left[\text{Fe}(\text{II})\text{Fe}(\text{II}){\left(\text{CN}\right)}_{6}\right]}^{2-}\left(\text{PW}\right)\\ \text{Fe}{\left(\text{III}\right)}_{3}\left[\text{Fe}\left(\text{III}\right){\left(\text{CN}\right)}_{6}\right]2\left[\text{Fe}\left(\text{II}\right){\left(\text{CN}\right)}_{6}\right]-\left(\text{BG}\right)\\ \left[\text{Fe}(\text{III})\text{Fe}(\text{III})(\text{C}{\text{N}}_{6}](\text{PY}\right)\\ \text{PB}+{\text{H}}^{+}+•\text{OH}\to \text{PY}+\;{\text{H}}_{2}\text{O}\\ {\text{H}}_{2}{\text{O}}_{2}\to {\text{O}}_{2}+2{\text{e}}^{-}+\;2{\text{H}}^{+}\\ {\text{H}}_{2}{\text{O}}_{2}+2{\text{e}}^{-}+2{\text{H}}^{+}\to \;2{\text{H}}_{2}\text{O}\\ \text{TMB}+{\text{H}}_{2}{\text{O}}_{2}+2\text{H}\overset{\text{PY}}{\to }\text{TMB}(\text{oxidized})+\;2{\text{H}}_{2}\text{O} \end{array}$$

In neutral and basic microenvironment, PB could show catalase-mimetic activity, which effectively accelerates the decomposition of H_2_O_2_ into H_2_O and O_2_. The reaction mechanism is as follows:

$$\begin{array}{l} \text{PB}+{\text{e}}^{-}\to \text{PW}\\ 3\text{PB}\to \text{BG}+\;2{\text{e}}^{-}\\ \text{PB}\to \text{PY}+{\text{e}}^{-}\\ 3\text{PB}+{\text{H}}_{2}{\text{O}}_{2}\to \text{BG}+\;2{\text{OH}}^{-}\\ 2\text{BG}+{\text{H}}_{2}{\text{O}}_{2}\to 6\text{PY}+\;2{\text{OH}}^{-}\\ 6\text{PY}+{\text{H}}_{2}{\text{O}}_{2}+2{\text{OH}}^{-}\to 2\text{BG}+{\text{O}}_{2}+\;2{\text{H}}_{2}\text{O} \end{array}$$

Generally, PB can be converted into Prussian White (PW) and oxidized into Berlin Green (BG) or Prussian Yellow (PY) at pH 3.0–8.0, which would then react with H_2_O_2_ to complete the redox cycle. When the electrode potential is below 0.7 V, high spin Fe^3+^/^2+^ plays the dominant role in the electron transfer process. Meanwhile, when the electrode potential is more than 0.9 V, [Fe(CN)_6_]^3−^/^4−^ becomes the dominant species. At an acidic pH, the H_2_O_2_ exhibits strong oxidation capabilities and effectively oxidizes the PB into BG or PY, resulting in the peroxidase-mimetic activity. It's also worth mentioning that the catalytic activity of PB nanoparticles are very sensitive to environmental pH, as the oxidative capability of H_2_O_2_ would become weaker under basic pH, leading to reduced peroxidase-like activity while enhancing the catalase-mimetic activity.

In another study by Luo et al. the authors developed a DOX-Fe^2+^ complex and loaded it into tumor-responsive amorphous calcium carbonate (ACC) nanostructures, which was further functionalized with PAMAM dendrimer with folate or MMP2-sheddable PEG. The DOX-Fe^2+^ complex could not only enhance the *in vivo* stability of the catalytically active Fe^2+^ ions, but also elevated the production of H_2_O_2_ by activating the NAPDH pathways to sustain the catalytic activity of Fe^2+^ ions, thus efficiently inducing ferroptotic cell death in cancer cells (Xue et al., 2020).

The catalase-mimicking properties of INs have also been explored to reduce hypoxia-induced resistance to cancer therapy. The feasibility and potentially therapeutic advantages of this strategy were demonstrated in the previous study, in which a hybrid nanosphere containing Fe^3+^ was introduced to overcome tumor hypoxia through decomposing endogenous H_2_O_2_. The hybrid nanosphere was constructed through the coordination-driven assembly of ferric ions, TPEDXX, and sabutoclax. Experimental results showed that once the hybrid nanospheres were taken in by cancer cells, the intracellular H_2_O_2_ would decompose into oxygen and enhance the Fe^3+−^catalyzed Fenton reaction. Meanwhile, sabutoclax could mitigate the PDT resistance through Bclx2 inhibition and enhance the ROS production under laser irritation (Shi et al., 2020).

It's also possible to synthesize iron-based nanocatalysts with tailored catalytic activities through the doping of other atoms. Typically, He and coworkers synthesized Fe-N-C artificial enzymes capable of activating oxygen for monooxygenation and dehydrogenation. The Fe-N_x_ center was an active site for O_2_ activation by directly producing specific reactive oxygen species (ROS). The O_2_ activation at the Fe-N site was caused by strong interactions between the N-doped carbon support and catalytic sites, which altered the electronic structure of Fe-N side. During the initiation of O_2_, the Fe-N site may bound and stimulate ^3^O_2_ to create ^1^O_2_, and then was modified to ${\text{HO}}_{2}{•}^{-}$ by receiving electrons and protons from other substances. Afterwards, the produced ${\text{HO}}_{2}{•}^{-}$ was converted to HO_2_- through the monooxygenation or dehydrogenation of organic substrates, which could effectively cause tumor cell death by elevating the ROS stress above the cytotoxic threshold. Interestingly, the N-doped nanomaterials also possessed high stability at extreme pH and are resistant to treatment with polar organic solutions (such as CH_2_Cl_2_, CH_3_CN, and *n*-hexene), heating (up to 70°C), and air exposure, which may be attributed to the robust structure of Fe-N-C (Tan et al., 2018).

## Critical Considerations for the Iron Nanozyme-Mediated Biocatalytic Tumor Therapy

Despite the excellent therapeutic performance of iron nanozymes demonstrated *in vitro* and *in vivo*, it should be recognized that a tumor is a highly complex disease and the efficacy of IN-mediated biocatalytic tumor therapy may be affected by a variety of issues in the clinical context. These limiting factors and notable issues would be discussed in the section below.

### Intratumoral H_2_O_2_ Level in Cancer Cells

As we have discussed in previous sections, most of the current iron nanozymes react with the endogenous H_2_O_2_ in the tumor cells to exert therapeutic effect. High local H_2_O_2_ level is a characteristic feature in many types of tumor indications due to the intense metabolism thereof. However, the endogenous H_2_O_2_ supply may also be exhausted or diminished due to various reasons and become a limiting factor for IN-mediated biocatalytic therapy (Huo et al., 2017). Therefore, it's sometimes necessary to incorporate supplementary measures to replenish the H_2_O_2_ in the tumor region to sustain the treatment (Ranji-Burachaloo et al., 2018).

In a recent study by Yeh et al. the authors encapsulated H_2_O_2_ into Fe_3_O_4_-PLGA polymersome to provide O_2_ for echogenic reflectivity as well as to sustain the •OH production. The Fenton reaction could not be triggered without ultrasound treatment as the Fe_3_O_4_ and H_2_O_2_ components were separated by the polymeric contents in the system. The ultrasound treatment could disrupt the polymersomes and potentiates the reaction between H_2_O_2_ and Fe_3_O_4_ nanoparticles to generate •OH via Fenton reaction. Moreover, because of the Fe_3_O_4_ content, the H_2_O_2_/Fe_3_O_4_-PLGA polymersome also allows for the magnetic resonance imaging of the tumor tissues (Li et al., 2016). In another study by Huo et al., the authors incorporated Fe_3_O_4_ and GOx into biodegradable dendritic silica nanoplatforms with large pore size to construct an H_2_O_2_ self-replenishable Fenton nanocatalyst for tumor therapy. GOx will convert the glucose in the tumor cells in the presence of oxygen and water to gluconic acid and H_2_O_2_, which would then be catalyzed by the Fe_3_O_4_ nanoparticles into highly toxic hydroxyl radicals in a sequential manner and eventually lead to the cancer cell apoptosis (Huo et al., 2017; Jiang et al., 2019, Figure 2).

**Figure 2:**
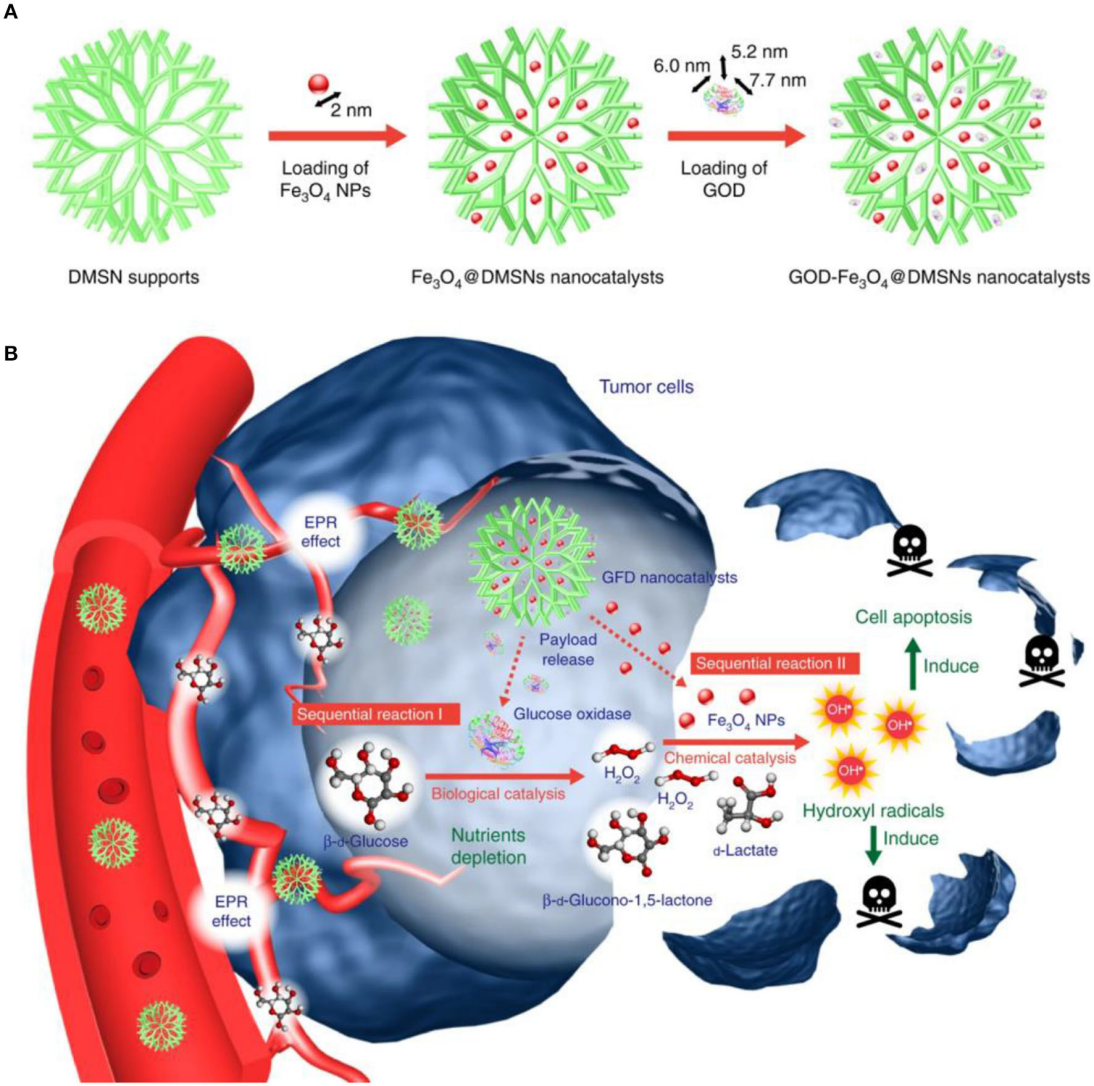
The scheme of sequential catalytic-therapeutic mechanism of GFD NCs on the generation of hydroxyl radicals for cancer therapy. **(A)** Synthesis process of the GFD NCs. **(B)** *In vivo* action of the GFD NCs. Reproduced with permission from Huo et al. (2017). Copyright © 2017, Springer Nature.

### Tumor Specificity

Intravenous injection is one of the primary methods for the administration of antitumor nanoagents, during which the nanoformulations would circulate around the body and eventually accumulate at the desired site of action. Nevertheless, after the nanoformulations enter the blood circulation, they may be captured and eliminated by the mononuclear phagocyte system, which would severely compromise their bioavailability and increase the risk of the undesirable nanoparticle accumulation in healthy organs. An alternative approach to address this issue is to conjugate long-circulating and targeting moieties to the nanoparticle surface (Dai et al., 2016). Generally, there are two types of targeted drug delivery: active targeting and passive targeting. In passive targeting, the cancer-targeting efficiency is closely correlated to physical features of nanocarriers such as size, hydrophilicity, and surface charge. In active targeting, the constructed nanocarrier could interact with targeting tumor cells to enhance the specific retention and uptake, which depends on the specific association between targeting ligand conjugated on the nanocarrier's surface and the receptors on the diseased tissues/cells surface (Dai et al., 2016). At the current stage, the functionalization of targeting ligand to the surface of INs is still rarely explored, which warrants more research input in the future.

### Optimization of the Catalytic Microenvironment

**Figure 3:**
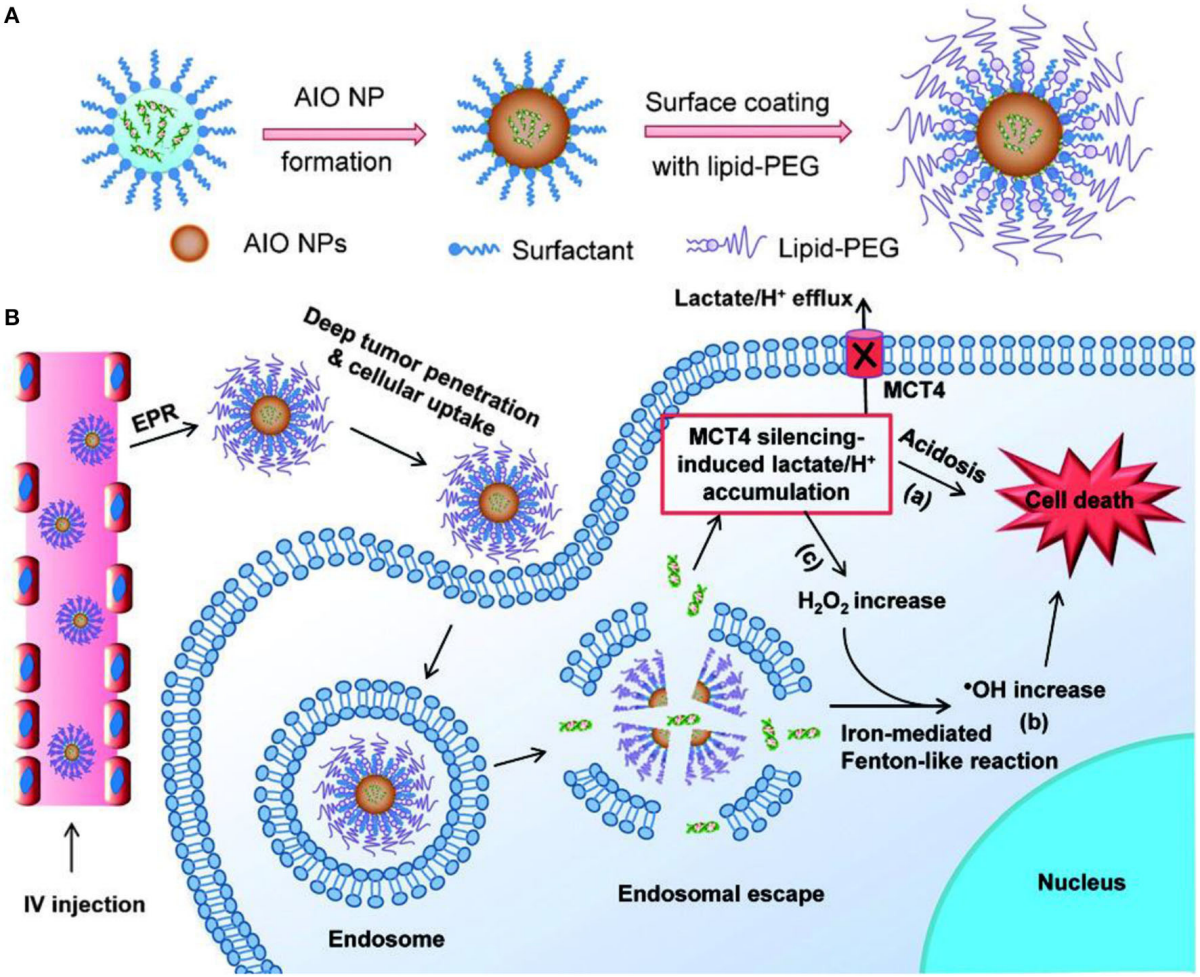
Schematic illustration of the AIOI RNAi NPs **(A)** and the mechanism of action **(B)** in the tumor microenvironment. Reproduced from Liu et al. (2018). Copyright © 2018 The Authors. Published by Wiley-VCH Verlag GmbH & Co. KGaA.

It's well-established that the catalytic functions of enzymes are highly susceptible to factors including pH, temperature, ionizable salts, and surfactants (Iyer and Ananthanarayan, 2008). Therefore, it's often necessary to optimize the reaction condition (acidity, temperature) of nanozymes in the biological environment to improve their efficacy. The optimal reaction pH for a Fenton reaction ranges from 2 to 4 due to the two major reasons: (i) it prevents the precipitation of iron ions under acidic environments and (ii) it inhibits degradation of the H_2_O_2_ under extremely low pH conditions. Meanwhile, the pH of tumor microenvironments range from 6.5 to 7.0, while the cancer cell endosomes have a pH of about 5.0 and lysosomes have a pH of ~4.5. Therefore, the strategy to reduce the pH of tumor microenvironments or deliver the drugs to the lysosome can effectively facilitate the Fenton reaction (Tang et al., 2019). For example, Liu and coworkers constructed an amorphous iron oxide-based RNAi-incorporated nanoplatform, which could effectively escape from endosome through osmotic pressure and enter the tumor cytosol (Liu et al., 2018). Subsequently, the co-delivered siRNA could inhibit the MCT4-mediated lactate efflux routes to acidify the intracellular environment (Figure 3) and thus enhance the Fenton-based catalytic efficacy. Moreover, the small size of the nanoplatform enabled them to penetrate the tumor tissues and enhance tumor deposition via EPR effect.

### Physicochemical Factors

It's well-known that the catalytic properties of nanozymes can be affected by their size, shape, concentration, structure, and chemical composition (Niu et al., 2010; Zhang et al., 2014). Therefore, there has been a growing interest in modulating the physicochemical properties of nanozymes to enhance their efficacy in a biological environment.

Particle size is one of the most important parameters that affects the catalytic activity of nanomaterials. It's widely accepted that nanozymes with smaller sizes usually have better catalytic activity than the larger counterparts, the size-induced changes in catalytic efficiency may be caused by the fact that small nanozymes have larger surface areas and a greater number of active sites (Könczöl et al., 2013; Li et al., 2015; Peng et al., 2015). In 2007, Yang et al. investigated the catalytic activity of Fe_3_O_4_ nanoparticles at different sizes (30, 150, and 300 nm). The experimental results indicated that 30 nm Fe_3_O_4_ nanoparticles exhibited the highest peroxidase-mimetic activity, while the 300 nm Fe_3_O_4_ nanoparticles showed the lowest catalytic ability (Gao et al., 2007). A similar trend has also been observed on Prussian blue nanoparticles, in which the researchers found that the catalytic efficiency of Prussian blue nanoparticles with an average diameter of 200 nm was 300 times higher than nanoparticles sized around 570 nm (Komkova et al., 2018).

Aside from nanoparticle size, shape and surface composition must also be considered when modulating the catalytic property of INs. Previous study revealed that between three Fe_3_O_4_ nanostructures, cluster spheres, octahedra, and triangular plates displayed varied peroxidase-mimetic catalytic efficacy, of which the cluster spheres were highest while octahedra ones were lowest. This may be caused by the preferential exposure of catalytically-active Fe crystal planes or atoms (Liu et al., 2011). Typically, nanomaterials have many facets with varying surface energies. Consequently, the catalytic potential of different facets may change greatly from one to another. The high-energy (110) facet tends to have a higher catalytic activity, which may be attributed to the open surface structure and greater number of active sites (Jiang et al., 2010; Liu et al., 2011).

The activity of the INS also could be enhanced by doping or integrating them with other materials, such as Ag, Au, and Pt. The peroxidase-mimetic activity of Au@Fe_3_O_4_ was higher compare to Au or Fe_3_O_4_ alone, which may attribute to the special electronic structure at the interfaces between them and the polarization effects from Au to Fe_3_O_4_. Other metals, such as Pt_48_Pd_52_-Fe_3_O_4_, Fe_3_O_4_@Pt, and Fe_3_O_4_ coated Ag nanowire also displayed the enhanced activity with higher stability compared to INs alone (Lee et al., 2010; Ma et al., 2013; Sun et al., 2013; Wang et al., 2016).

### Biosafety/Biocompatibility/Biodegradability

Aside from the promising antitumor efficacy of iron-based nanozymes, it's also necessary to investigate their potential impact on the health of the receiving host both in the short term and long term. Consequently, the INs should be non-toxic to the normal tissues and preferably be degraded in biological environments and eventually eliminated from the body after completing the therapeutic events. Biocompatibility and biosafety of both enzymes and nanozymes play the key role in ensuring their further clinical application. Nevertheless, INs are still a new technology in the early stage of development and most of their interaction patterns with the biological environment remain to be elucidated, which warrants continuous research input regarding their pharmacokinetics, absorption, distribution, metabolism, therapeutic sustainability, excretion, and toxicity at molecular, cellular, and systemic levels (Tibbitt et al., 2016).

### Stimulation by External Irritation

The regeneration of Fe^2+^ species in cancer cells is also a limiting factor that affects the treatment-induced ROS stress. The photo-Fenton reaction is an emerging strategy to accelerate the regeneration of Fe^2+^ ions using external irradiation, which could significantly enhance the generation efficiency of hydroxyl radicals comparing to classical Fenton catalysts. From a mechanistic perspective, the Fe^3+^ species in the solution could undergo an efficient photoreaction under light illumination to generate Fe^2+^ and additional hydroxyl radicals, thus potentiating improved tumor inhibition effect. For example, the photo-Fenton strategy has been applied to enhance the catalytic efficiency of TAT peptide-conjugated Fe_3_O_4_ nanoparticles in tumor microenvironments. The results suggested that the combinational treatment of Fe_3_O_4_-TAT nanoparticles and 5 Gy radiation therapy effectively prevents tumor growth (Ranji-Burachaloo et al., 2018). These discoveries hold a great promise for improving the biocatalytic activity of INs against cancer for clinical translation.

## Future Perspective and Current Challenges

Although nanozymes have demonstrated great potential for tumor therapy, there are still some issues that need to be resolved for broad clinical implementation. First of all, unlike natural enzymes, most iron-based nanozymes have a low catalytic specificity and could respond to multiple substrates in the biological milieu, and this poor substrate selectivity may interfere with the endogenous enzyme-mediated catalytic pathways and increase the risk of undesirable side effects. Therefore, it's of high urgency and importance to investigate the overall impact of nanozymes on the catalytic landscape *in vivo* and improve their substrate selectivity. Secondly, the catalytic mechanisms of existing INs are still under investigation, and comprehensive mechanistic investigations are still needed, which could not only explain the correlation between the catalytic performance and the composition/structure of the nanozymes, but also provide indicative insight for the development of novel nanozyme-based therapeutics. Furthermore, as most of the iron-based nanozymes are intrinsically multifunctional and may offer other benefits such as cancer treatment, imaging and sensitization, it's important to balance their catalytic activity and other functions through rational structural design and surface modification. Finally, a thorough investigation is still needed to investigate the biosafety of these nanozymes in clinically relevant models, which may fill the blank in current nanozyme research and greatly facilitate their clinical translation.

## Conclusion

To sum up, the intrinsic enzyme-mimetic activity of INs has been exploited for decades. As the new generation of artificial enzymes, the catalytic behaviors of INS have been systematically analyzed and improved by adjusting certain parameters, such as optimizing their physicochemical properties or modulating their local environment. Compared to natural enzymes, INs displayed higher stability and modification versatility, which opens up new avenues for nanocatalytic tumor therapy. However, several challenges still hinder its clinical application, which should be investigated in the future by both scientists and biomedical researchers.

## Author Contributions

ZL, ML, and YHo conceptualized this review. LS and ML wrote the paper and prepared the figures. All authors revised the manuscript.

## Conflict of Interest

The authors declare that the research was conducted in the absence of any commercial or financial relationships that could be construed as a potential conflict of interest.
